# Reliability and validity assessment of a survey: Measuring satisfaction with cochlear implant rehabilitation services for children in Jordan

**DOI:** 10.1371/journal.pone.0295939

**Published:** 2023-12-18

**Authors:** Rana Alkhamra, Hala M. Al-Omari, Hanady A. Bani Hani

**Affiliations:** 1 Department of Hearing and Speech Sciences, Faculty of Rehabilitation Sciences, The University of Jordan, Amman, Jordan; 2 Department of Hearing and Speech Sciences, School of Rehabilitation Sciences, The University of Jordan, Amman, Jordan; Jordan University of Science and Technology, JORDAN

## Abstract

**Background:**

Assessing parental satisfaction with healthcare services is crucial, particularly for parents and their children, who are the primary recipients of these services. In the context of Arabic-speaking parents, there is a notable absence of survey instruments tailored to measure their satisfaction. This study seeks to address this gap by validating a survey designed to evaluate parental satisfaction with rehabilitation services (RSs) provided to Jordanian children who have received cochlear implants (CIs).

**Methods:**

The study included 92 participants and followed a four-step methodology: 1) a literature review and expert input; 2) cognitive interviews, pilot testing, and test-retest reliability testing; 3) data collection; 4) validity and reliability assessments.

**Results:**

The survey’s validity was confirmed. Expert input and cognitive interviews improved content validity, and factor analysis established construct validity by revealing six factors explaining 82.33% of the variance in the survey scale. Convergent and discriminant validity were confirmed (composite reliability >0.7 and average variance extracted value >0.5). Cronbach’s α exceeded 0.8 for each factor and reached 0.855 for the total scale. Survey results showed reliance on speech therapy and audiology, varied rehabilitation durations, and progress. Parents expressed overall satisfaction, particularly influenced by technical quality and efficacy/outcome dimensions. Parents’ recommendations to enhance satisfaction with RSs included financial support, improved service accessibility, enhanced service delivery, specialized education, and increased public awareness.

**Conclusion:**

This study validates an Arabic satisfaction survey, emphasizing the significance of multidisciplinary, extended rehabilitation programs, skilled professionals, and positive outcomes. It emphasizes the necessity for improved access to specialized care and collaboration among healthcare, government, and media to shape parental perceptions of RSs. While the findings indicate overall satisfaction, they also reveal challenges faced by parents, highlighting the need for comprehensive support systems. These insights assist healthcare providers and policymakers in enhancing care quality and meeting the needs of CI children’s families, thereby improving the RSs experience in Jordan.

## Introduction

Hearing loss (HL) is the world’s fourth-leading cause of disability [1]. Without access to early diagnosis and appropriate intervention or rehabilitation, children with HL may have lifelong deficits in speech and language acquisition, poor academic performance, social maladjustments, and emotional difficulties [2, 3]. Hence, having an effective healthcare system that considers the entire spectrum of HL care, from diagnosis to rehabilitation, as well as thoroughly evaluating that system to enhance the quality of care, is crucial [4].

To enhance the quality of care, pediatric studies have focused on assessing the effectiveness of the healthcare system by examining parental attitudes [5]. Since parents and their children are the primary recipients of healthcare services, measuring parental satisfaction serves as a crucial indicator of performance and outcomes, forming an integral part of quality assessment for these services [6]. Parental satisfaction is defined as "an attitude about service, service providers, or patients’ health status" [7]. Through expressing satisfaction or dissatisfaction, parents can gauge the quality of services provided and shed light on their strengths and weaknesses, thus offering opportunities to identify and address service gaps [8]. Typically, a questionnaire is used to assess parental satisfaction [9].

Numerous studies have demonstrated the influence of various factors on parental satisfaction with rehabilitation services (RSs). These factors encompass early detection and intervention of HL [10, 11], as well as the efficacy and quality of rehabilitation in shaping auditory, speech, and language skills development [10, 12–15]. Additionally, accessibility to rehabilitation facilities and the attitudes and expertise of professionals delivering services within these facilities play a crucial role [15].

Moreover, it is imperative to consider other factors that come into play. These include the availability of personal and emotional support, as well as access to information provided by service providers following a child’s HL diagnosis [11, 14, 16, 17]. Financial aspects, such as the cost associated with rehabilitative services, have also been shown to impact parental satisfaction [15, 18].

With the advancements in cochlear implants (CIs), the majority of the literature on parental satisfaction with healthcare services and their outcomes for children with significant HL has been narrowed to this technology [5, 19]. A CI is a medical treatment option that enhances the treatment and prognosis of children with severe to profound sensorineural HL. Common RSs that follow receiving a CI involve auditory, language, and speech services [20]. While not all children with CI necessarily experience concurrent comorbidities, it is recognized that some children may have additional conditions or difficulties beyond their HL [21]. These concomitant comorbidities may include visual or cognitive impairments, sensory deficits, motor difficulties, emotional and behavioral issues, as well as other complexities [21]. To meet to the unique needs of these children, speech and hearing services are expanded to entail collaboration with other rehabilitation professionals such as occupational therapists, psychologists, and other specialized providers [22–25]. Accordingly, adopting a multidisciplinary approach in both the assessment and intervention for these children is critical.

A CI has been associated with increased parental satisfaction and progress in performance in the areas of listening (e.g., speech perception), communication (e.g., receptive and expressive language, speech, and voice clarity), interpersonal interactions and relationships (e.g., interactions with family members and others), and learning (e.g., academic performance, ability to perform multiple tasks, and attention) [13, 19, 26]. CI outcomes in these areas have been the focus of several studies examining parental satisfaction post-implant [13, 19, 26–28]. The majority of these investigations employed questionnaires to investigate whether parents’ pre-implant expectations for post-implant outcomes were met, and they consistently found that parents’ expectations were either met or surpassed.

Additionally, the duration of the rehabilitation process, and the type of insurance coverage for rehabilitation have all been linked to better CI outcomes and greater parental satisfaction with CIs [29–35]. Notably, the effects of the duration of rehabilitation on speech, language, and auditory skill development have been emphasized. For example, a study involving 15 children who received CIs at an average age of 45.27 months and underwent 8 months of aural rehabilitation approached but did not quite reach the developmental milestones for auditory, language, and speech skills [32]. These findings highlight the importance of long-term aural rehabilitation in accelerating and achieving age-appropriate developmental skills, ultimately contributing to higher levels of parental satisfaction with CIs.

The effects of the insurance type have also been the subject of investigation. Numerous studies, for instance, have suggested that having public health insurance, lower socioeconomic status, and parents with lower education levels can act as barriers to early implantation (i.e., before age 2) and achieving the best outcomes with the CI device [34, 36]. In contrast, other studies have identified private insurance as a barrier to the implantation process, presumably due to the higher cost of the insurance and associated deductibles compared to public health insurance [37]. Ultimately, regardless of whether it’s private or public insurance, research indicates the importance of access to RSs with appropriate health insurance coverage and financial assistance post-implantation. Such access has been shown to alleviate parental stress and contribute to higher levels of satisfaction with CI outcomes [38].

Other studies focused on CI have linked parental satisfaction with RSs to several key factors. These factors include the presence of local therapy support and receiving positive attitudes, guidance, and information from experts throughout the cochlear implantation process [39–41]. Conversely, Zaidman-Zait and Most (2005) found that mothers of children with CIs who experienced communication difficulties with rehabilitation professionals pre- and post-implant reported higher levels of stress and lower levels of satisfaction with RSs [33]. Furthermore, research investigating the support needs of parents of children with CIs has emphasized the positive impact of psychosocial supports, such as parent-to-parent support and participation in parent groups, on parental satisfaction with RSs [40, 42].

Despite the increasing prevalence of HL in Jordan, where 1.5% of Jordanian infants are born with mild to profound HL, there is a remarkable gap in the literature regarding rehabilitation studies focused on Arab countries, specifically Jordan. This percentage of infants with HL (i.e., 1.5) has been documented in a cross-sectional study involving 63,040 newborns [43]. In this study, 7% [63,41] of the infants initially failed the hearing screening and required retesting. After undergoing Auditory Brainstem Response tests, 966 out of 1103 (25.0%) infants were diagnosed with HL, with 590 experiencing sensorineural, 311 conductive, and 65 experiencing mixed HL. The severity of HL ranged from mild to profound, encompassing 182 infants with mild, 320 with moderate, 195 with severe, and 269 with profound HL [43].

The first CI surgery for deaf children was conducted in Jordan in 2003 [44]. In 2014, Hearing Without Borders (HWBs), an initiative that aimed to treat HL by providing deaf children with CI and the necessary medical and rehabilitative care, was launched throughout Jordan [44]. As a result of this initiative, CI had grown to a total of 1,107 children by July 2019 [45]. Despite the increasing prevalence of CI in Jordan and the significance of evaluating the CI process, there is a paucity of research in this area. To the best of our knowledge, only one study has examined the CI process in Jordan. In this study, Alkhamra (2015) looked into parents’ perceptions of the pre-implant process and whether their post-implant expectations were met. Sixty parents of deaf children participated in the study and completed a non-standardized, study-specific questionnaire [46]. The results indicated that parents were generally satisfied with the quantity and quality of pre-implant information they received, primarily from otolaryngologists. They also emphasized the importance of implementing a multidisciplinary team approach pre and post cochlear implantation.

The development and utilization of instruments for measuring patient satisfaction with healthcare services have been extensively explored in both English and non-English contexts. Notable examples include the Patient Satisfaction Questionnaire and the EUROPEP questionnaire for patient evaluation of general practice care [47, 48]. However, in the realm of Arabic healthcare services, especially within the domain of RSs, there is a paucity of research in the development of suitable assessment tools. Even when such tools have been employed, they are often translations from English and are primarily designed for family-centered services, which are not yet widely implemented in Jordan [49].

Additionally, it is essential to recognize the profound impact of diverse backgrounds, cultures, and contextual factors on the rehabilitation process [50]. Therefore, rehabilitation studies conducted in developed countries or even developing countries that do not speak Arabic can have different experiences and results from those conducted in Arabic-speaking countries. Consequently, using rehabilitation assessment instruments sourced from different languages, backgrounds, cultures, and contexts may not be suitable for evaluating RSs among Arabic-speaking individuals. Moreover, there is a notable absence of existing rehabilitation assessment questionnaires that have been applied specifically to Arabic-speaking parents of children with CIs.

To address this gap, we developed and validated a survey instrument tailored exclusively for this study’s purpose. This study has two main objectives: Firstly, it aims to validate the satisfaction scale integrated into the survey. Secondly, it seeks to investigate parental satisfaction with RSs for children with CIs in Jordan, focusing on four key objectives:

- To explore the RSs utilization patterns by children with CIs in Jordan.
- To determine the extent to which parents of children with CIs in Jordan express satisfaction with the RSs they receive.
- To determine which specific variables related to satisfaction are most strongly correlated with the overall satisfaction of parents of children with CIs with RSs.
- To highlight parents’ suggestions on how to enhance their satisfaction with RSs for children with CIs.

## Methods

### Procedures

The study aimed to design an Arabic survey to evaluate parents’ satisfaction with RSs. To do so, four steps were carried out: (1) a literature review and the design of the survey instrument; (2) cognitive interviews, piloting, and reliability testing; (3) determining sample size and construct validity testing; and (4) analysis of the evidence of convergent and discriminant validity. The stages are described below.

**Step 1**: *A literature review and the design of the survey*. The first step in the development of the survey instrument was to create items that delve into a comprehensive exploration of the study’s research objectives. Despite the fact that there is no consensus on the central content of a satisfaction questionnaire in healthcare, reviewing the literature indicated that there are certain satisfaction dimensions that are commonly investigated in healthcare satisfaction studies. Eight fundamental dimensions of satisfaction have been identified in Ware et al.’s (1977) systematic review, which is cited in Keith’s (1998) literature review. These dimensions include interpersonal manner (e.g., concern, consideration, friendliness, patience; negative aspects: causing embarrassment, disrespect), provider and facility availability, service accessibility/convenience (e.g., ease of making appointments, waiting time, convenience of location), technical quality (e.g., competence of providers, adherence to high standards of diagnosis and treatment), efficacy/outcomes (e.g., perceptions of the usefulness of care in maintaining or improving health status), continuity of care (e.g., regularity of care from the same provider or facility), physical environment (e.g., physical facilities, including cleanliness and comfort of accommodations), and financial aspects (e.g., ability to pay, payment mechanisms) [7, 9]. These dimensions or variables, in addition to discussions with parents of children with CIs and our own views, served as the foundation for the survey’s questions. The survey was structured into four sections:

*Section 1*, titled **‘Utilization Patterns of RSs,’** aimed to collect essential background information regarding the utilization patterns of RSs. *Section 2*, titled ‘**Satisfaction Variables**,’ focused on assessing satisfaction with rehabilitation by investigating six of the satisfaction dimensions identified by Ware et al. (1977). *Section 3*, titled ‘**Participant Characteristics**,’ explored parent and child characteristics, providing valuable contextual information. *Section 4*, titled ‘**Parents’ Suggestions**,’ examined parents’ views on how to enhance satisfaction with RSs. After the initial draft of the instrument was developed, the face and content validity of all sections’ items were reviewed by two highly qualified experts specializing in working with children with HL using CIs—a speech therapist and an audiologist, both holding doctoral degrees and possessing extensive experience in instrument design and validation. Furthermore, an Arabic language specialist, with a master’s degree in Arabic linguistics and literature and over a decade of experience teaching Arabic in educational settings, reviewed the questionnaire’s syntactic structures and semantics. The final version of the survey was then completed, incorporating all of the recommendations and insightful insights provided by the experts. Finally, the experts agreed that the survey’s items were appropriate.

**Step 2**: *Cognitive interviewing*, *piloting*, *and reliability testing*. After the survey’s design phase, which involved expert input, it underwent cognitive interviews and pilot testing. Cognitive interviews are employed in pre-testing a survey to assess if its questions and responses can accurately convey the intended meaning to respondents [51]. This method has become an essential step in developing standardized measures and serves as a source of validity [52].

Cognitive interviews were conducted with 15 participants. Based on the valuable feedback gathered during these interviews, the questions that were identified as problematic were revised. For instance, in *Section 2* of the survey question 7 originally asked whether parents were satisfied with the amount of information provided by service providers in the rehabilitation facility about their children’s hearing condition. Respondents suggested expanding the definition of "facility" to include "hospital, clinic, and center." Consequently, we incorporated these clarifications into the question. Furthermore, taking into account participants’ input, we decided to remove two questions focused on service providers’ "sincerity" and "friendliness." Participants noted that these questions seemed redundant in light of the scale item that asked about parents’ satisfaction with the consideration and compassion of rehabilitation providers toward children with CI and their caregivers. This led to a reduction in the total number of scale items from 20 to 18.

Following the revision of the problematic survey items, we conducted piloting with 30 participants with children with HL [53]. The calculation of Cronbach’s alpha, which measures the internal consistency or the extent to which similar questions produce consistent responses [54], yielded a value of 0.833 for *Section 2*. This result suggests a high level of internal consistency. Most of the items in *Section 2* demonstrated sufficient reliability. However, two specific items, namely "Satisfaction with the ease of finding rehabilitation providers" and "Satisfaction with the ease of making an appointment at the rehabilitation clinic or center," could potentially be removed to elevate the alpha to 0.840. Nevertheless, we decided to retain these items. The fact that the initial Cronbach’s alpha values were considered acceptable and that removing these items would not significantly increase alpha influenced this decision. Furthermore, because the number of scale items is relatively small, each item contributes valuable information to the survey.

To evaluate the test-retest reliability, we administered the same survey to the participants who had initially completed it during the pilot study, with a two-week interval between the two administrations. We assessed reliability using the intra-class correlation coefficient (ICC) with a corresponding 95% confidence interval. Our criterion for acceptability in terms of test-retest reliability was set at an ICC value of ≥ 0.70, adhering to predefined criteria [55].

**Step 3**: *Sample size and construct validity*. Following statisticians’ advice to use a survey sample size of 10% of the population as long as it doesn’t exceed 1,000 participants [56], the study’s participant count was determined. According to the Times newspaper’s most recent available statistical data, by 2019, about 1,000 children in Jordan had received CIs [45]. Consequently, it was deemed appropriate to limit the sample size to a maximum of 100 individuals for this study. To account for non-response rate during the survey data collection, 200 questionnaires were distributed to potential participants.

A non-probability convenience sampling method was utilized in data collection. This approach is commonly used in exploratory research to investigate the attitudes and perspectives of participants [57]. The inclusion criteria involved parents of congenital prelingually deafened children who had received CIs and had experience with RSs. Additionally, participants needed to be proficient in reading and writing standard Arabic. Excluded from the study were parents of children who had received CIs in Jordan but were receiving RSs in another country at the time of the study. Participants were recruited from various sources, including public speech and hearing clinics in hospitals and universities, private speech and hearing clinics, special education centers, and through personal contacts made by the researchers.

The study was approved by the Research and Ethics Committee at the University of Jordan (Decision No. (107–2022)). All the participants consented to participate in the study by signing informed consent form and completing and returning the survey. All methods were carried out in accordance with relevant guidelines and regulations. The survey was distributed alongside a cover letter that outlined the study’s objectives, stressed the voluntary nature of participation, and guaranteed the confidentiality of all personal information and opinions collected. To ensure anonymity, each participant was assigned a unique survey code, and they had the flexibility to complete the survey either during their facility visit or on a subsequent visit. Reminders and replacement copies were provided for those who didn’t return the survey. Participants were instructed to contact the study’s principal investigator via email or phone if they had any questions. Data collection spanned six months.

**Fig 1:**
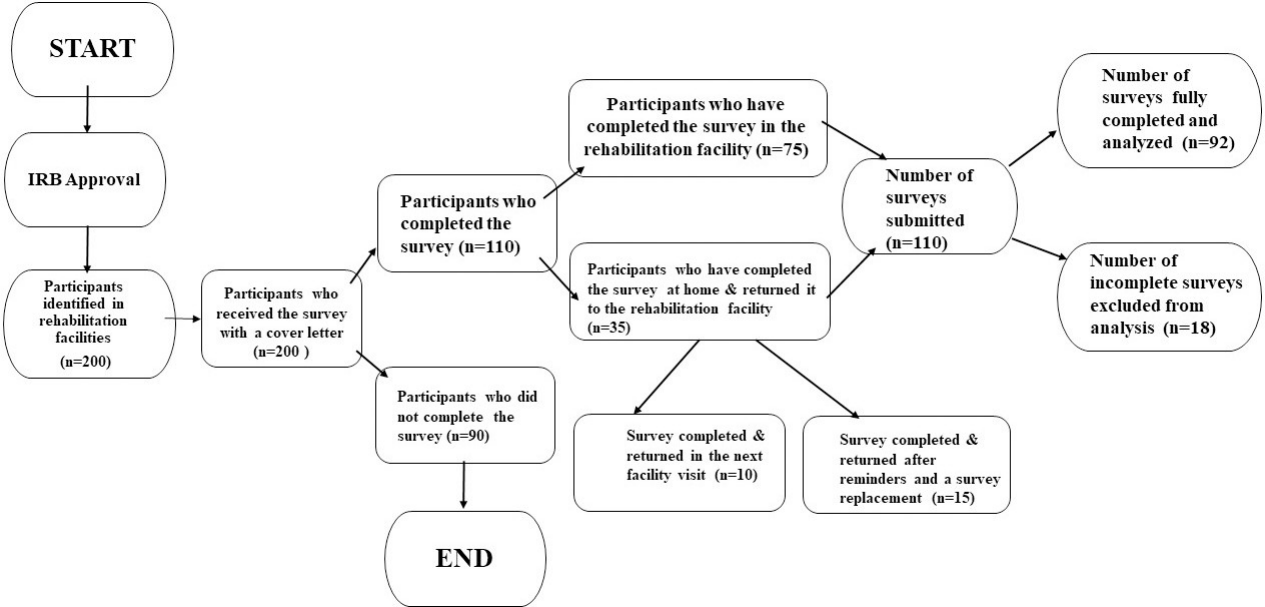
Survey data collection workflow: Sequential steps in the data gathering process.

Of the 200 surveys that were sent out, 100 (50%) were returned, and 92 (46%) were used in the analysis. It’s worth noting that eight surveys had to be excluded from the analysis due to significant missing data. The participants who chose not to return the survey cited reasons such as changing their minds about study participation, switching therapy facilities, or discontinuing therapy. Fig 1 illustrates a flowchart detailing the process of data collection.

The 92 participants’ data was used to conduct an exploratory principal component factor analysis on the survey’s *Section 2* scale questions. Varimax (orthogonal) rotation was used for extracting factors using eigenvalues and determining factor loadings. Consistent with comparable studies methodologies, items with factor loading scores exceeding 0.5 for their contribution to the factors were retained [58]. This statistical procedure was implemented to confirm the six-factor construct of the scale.

**Step 4**: *Convergent and discriminant validity*. Convergent and discriminant validity were assessed to determine whether the scale accurately measured the dimensions intended. Three criteria were used to evaluate convergent validity. First, the factor weight of each item within its respective factor had to be greater than 0.50 [59]. Second, the composite reliability index needed to be above 0.70 [59]. Third, the average variance extracted (AVE) had to surpass 0.50 [60].

### Instrument

The final survey consisted of four sections, a total of 39 questions, and a variety of question formats. In particular, *Section* 1, titled ‘**Utilization patterns of RSs**,’ and *Section 3*, titled ‘**Participant characteristics**,’ consisted of closed-ended multiple-choice questions; *Section 2*, titled **‘Satisfaction variables,’** utilized a 5-point Likert scale; and *Section 4*, titled ‘**Parents’ suggestions**,’ featured an open-ended question. *Section 1* gathered information about the types of RSs commonly received, the duration of rehabilitation, the progress in performance post-rehabilitation, and overall satisfaction with RSs. *Section 2*, comprising 18 items, used a 5-point Likert scale response format ranging from extremely dissatisfied = 1 to extremely satisfied = 5 to assess satisfaction across the six RS dimensions: 1. efficacy and outcome; 2. financial aspects; 3. technical quality; 4. availability of service providers; 5. accessibility and convenience; and 6. personal manner. For a detailed breakdown of the questions and responses in *Sections 1* and *2*, please refer to Table 1.

**Table 1:** *Sections 1* and *2* items and their corresponding responses.

	***Section 1*- Multiple choice questions**						
	**Items**	**Responses**					
Q1	What types of rehabilitation services have your child regularly received or is currently receiving?	Audiology	Speech therapy	Occupatio-nal Therapy	Physical Therapy	Psycholo-gy	Other
Q2	For how long has your child been receiving rehabilitation services?	≤ 6 mos	7 mos– 1;0 yr.	1;1–2;0 yr.	2;1–3;0 yr.	3;1–4;0 yr.	>4 yr.
Q3	How do you describe the progress in your child’s performance following receiving rehabilitation services?	Significant progress	Acceptable progress	Limited progress	I can’t decide	No progress	
Q4	How would you rate your overall satisfaction with the rehabilitation services your child has received?	Extremely Dissatisfied	Dissatisfied	Neither dissatisfied or satisfied	Satisfied	Extremely satisfied	
	***Section 2*—Five-level Likert satisfaction questions**						
	**Responses: 1**. **Extremely dissatisfied**; **2. Dissatisfied**; **3**. **Neither dissatisfied or satisfied**; **4. Satisfied**; **5. Extremely satisfied**						
**Item no**.	**How satisfied or dissatisfied are you with the following?**	**1**	**2**	**3**	**4**	**5**	
7	The amount of information provided by rehabilitation providers in the clinic/hospital/center about your child’s hearing condition.						
11	The overall effectiveness of therapy						
12	The effect of therapy on the growth of your child’s developmental skills.						
13	The rate of developmental skills growth attributable to therapy.						
14	The guidance provided by rehabilitation providers on how to continue therapy at home.						
16	Health insurance services						
15	The cost of rehabilitation services						
17	The extent to which rehabilitation expenses are covered by health insurance.						
8	The adequacy of information provided about available resources and services for a child with a CI.						
5	The evaluation process pre-implant						
6	The timing of the diagnosis of hearing loss and its impact on the developmental skills of the child.						
9	The act of referring your child to the relevant specialists when necessary.						
1	The ease of finding rehabilitation providers						
3	Satisfaction with the ease of locating a rehabilitation clinic/center						
2	The ease of making an appointment at the rehabilitation clinic/center						
4	The accessibility of the rehabilitation center/clinic or specialist’s location.						
10	The consideration and compassion exhibited by rehabilitation providers towards children with CI and their caregivers.						

* Note: The items in *Section 2* were assigned numbers in the table based on their position within the scale.

*Section 3* encompassed participant characteristics, providing valuable contextual information. Tables 2 and 3 present an overview of child and parental characteristics, respectively. Lastly, *Section 4* features an open-ended question to collect suggestions for improving parental satisfaction with RSs for children with CIs in Jordan. This diversified survey structure ensured a comprehensive exploration of the research objectives, enhancing the depth of our data collection.

**Table 2:** Child characteristics.

Child characteristics	n(%)	Child characteristics	n(%)
**1. Age (year; month)**		**6. Age at receiving a CI (year; month)**	
≤ 1;0	16(17.4)	≤ 1;0	28(30.4)
1;1–2;0	6(6.5)	1;1–2;0	12(13.0)
3;0–4;0	26(28.3)	3;0–4;0	38(41.3)
5;0–12;0	44(47.8)	5;0–12;0	13(14)
> 12;0	0	Missing	1(1.1)
Missing	0	**7. Mode of communication**	
**2. Gender**		Spoken language	40(44.0)
Male	62(67.4)	Lip/speech reading	7(7.7)
Female	30(32.6)	Spoken & sign language	21(23.1)
Missing	0	Spoken language & speech reading	9(9.9)
**3. Onset of hearing loss**		Total communication	14(15.4)
Congenital	47(51.1)	Missing	1(1.1)
Sudden	31(33.7)	**8. Comorbidities associated with HL**	
Progressive	5(5.4)	Yes	21(22.8)
Missing	9(9.8)	No	69(75.0)
**4. Age at HL diagnosis (year; month)**		Missing	2(2.2)
≤ 1;0	52(56.5)	**9. Order in the family**	
1;1–2;0	22(23.9)	Youngest	23(25.0)
3;0–4; 0	15(16.3)	Middle	38(41.3)
5;0–12;0	0	Eldest	23(25.0)
Missing	3(3.3)	Other	4(4.3)
**5. Hearing device used**			
Unilateral CI	78(84.8)		
Bilateral CIs	8(8.7)		
CI+HA	6(6.5)		
Missing	0		

**Table 3:** Parent characteristics (n = 92).

Parent characteristics	n(%)	Parent characteristics	n(%)
**1. Age (year)**		**5. Health insurance coverage**	
< 20	2(2.2)	Public	31(33.7)
21–30	16(17.4)	Private	41(44.6)
31–40	46(50.0)	No health insurance	19(20.7)
41–50	26(28.3)	Missing	1(1.1)
> 51	2(2.2)	**6. Residence**	
Missing	0	East Amman	35(38)
**2. Education**		West Amman	16(17.4)
< High school	30(32.6)	Outside Amman	39(42.4)
High school	22(23.9)	Missing	2(2.2)
College/diploma	25(27.2)	**7. Yearly income**	
Bachelor degree	7(7.6)	≤ 4000 JD	66(71.7)
Master’s degree	2(2.2)	5000–10000 JD	16(17.4)
PhD degree	0	11000–20000 JD	7(7.6)
Missing	6(6.5)	21000–30000 JD	0
**3. Number of children in the family**		> 30000 JD	0
1 child	10(10.9)	Missing	3(3.3)
2–3 children	32(34.8)	**8. Have multiple children with HL**	
4–5 children	38(41.3)	Yes	35(38)
6–7 children	12(13)	No	53(57.6)
>7 children	0	Missing	4(4.3)
Missing	0		
**4. Parents are blood related**			
Yes	61(66.3)		
No	29(31.5)		
Missing	2(2.2)		

## Data analysis and results

### Validity and reliability of the scale

#### Validity

*Content validity*. After the survey was designed with the support of experts, content validity was enhanced by conducting cognitive interviews. Conducting cognitive interviews helped ensure that the questionnaire questions were clear, relevant, and comprehensible to the target population, which in turn enhanced the content validity of the survey items.

*Construct validity*. *Section 2* of the survey, consisting of 18 scale question items, underwent an exploratory factor analysis to assess its construct validity (n = 92). The suitability of the data for factor analysis was assessed through the Kaiser-Meyer-Olkin (KMO) measure and Bartlett’s Test of Sphericity. Our results indicated that the sample was factorable (KMO = 0.71) and Bartlett’s test of sphericity was significant (χ^2^(153) = 804.124, p < 0.001). Results from the principal component analysis revealed the presence of six factors, as indicated by eigenvalues, collectively explaining 82.33% of the variance. Specifically, the eigenvalues for these six factors—1) efficiency and outcome, 2) financial aspects, 3) technical quality, 4) service provider and facility availability, 5) service accessibility and convenience, and 6) interpersonal manner—explained 35.324%, 16.144%, 9.716%, 7.882%, 7.372%, and 5.889% of the variance, respectively.

To rotate the six factor components, we employed a varimax (orthogonal) rotation. Table 4 presents the loadings of each item on its respective factor. Items with factor loadings exceeding 0.5 were retained in the final rotated solutions [59]. One item about participants’ “overall satisfaction with rehabilitation services” presented a challenge by cross-loading on two factors. To address this issue, we followed the recommendation that proposed removing the item when it exhibits high loadings (≥ 0.5 in our study) on two distinct factors, and the difference between these factor loadings is less than 0.10 [61, 62]. Consequently, we decided to exclude this item from the Likert scale questions in *Section 2* of our research, leaving a total of 17 remaining items in this section. Instead, we relocated this item to *Section 1*. We made this adjustment because the item provides valuable insights into participants’ overall satisfaction, which is crucial for our study’s analysis. For a comprehensive breakdown of the remaining items and their respective loadings on their respective factors, please refer to Table 4.

**Table 4:** The Likert scale items in *Section 2* are organized according to their loadings on the satisfaction dimensions, as determined through principal component analysis.

	Satisfaction dimensions						
	1. Efficacy and outcome; 2. Financial aspects; 3. Technical quality; 4. Availability of service providers; 5. Accessibility and convenience; 6. Personal manner						
‘Item no.	Satisfaction scale items	1	2	3	4	5	6
7	Satisfaction with the amount of information provided about the child’s hearing condition	0.718					
11	The overall effectiveness of therapy	0.796					
12	Satisfaction with the effect of therapy on the growth of your child’s developmental skills.	0.843					
13	Satisfaction with the rate of developmental skills growth attributable to therapy.	0.851					
14	Satisfaction with the guidance provided by rehabilitation providers on how to continue therapy at home.	0.683					
16	Satisfaction with health insurance services		0.892				
15	Satisfaction with the cost of rehabilitation services		0.933				
17	Satisfaction with the extent to which rehabilitation expenses are covered by health insurance.		0.923				
8	Satisfaction with the adequacy of information provided about available resources and services for a child with a CI.			0.806			
5	Satisfaction with the evaluation process pre-implant			0.805			
6	Satisfaction with the timing of the diagnosis of hearing loss and its impact on the developmental skills of the child.			0.905			
9	Satisfaction with the act of referring your child to the relevant specialists when necessary.			0.625			
1	Satisfaction with the ease of finding rehabilitation providers				0.945		
3	Satisfaction with the ease of locating a rehabilitation clinic/center				0.948		
2	Satisfaction with the ease of making an appointment at the rehabilitation clinic/center					0.901	
4	Satisfaction with the accessibility of the rehabilitation center/clinic or specialist’s location.					0.905	
10	Satisfaction with the consideration and compassion exhibited by rehabilitation providers towards children with CI and their caregivers.						0.910

*Convergent validity*. Table 5 shows the results of convergent validity. The average extracted variance (AEV) was higher than 0.5 [59]. Additionally, the composite reliability (CR) was higher than 0.7, which is a positive indicator [63]. Thus, we conclude that the questions effectively assess the constructs established in each factor and demonstrate convergent validity.

**Table 5:** Convergent and discriminant validity analysis results.

	Convergent validity analysis			Discriminant validity analysis
Dimensions of satisfaction	Average loading	AVE	Composite reliability (CR)	
1. Efficacy/Outcome	0.766	0.586	0.878	
2. Financial aspects	0.91	0.827	0.935	
3. Technical quality	0.74	0.547	0.863	
4. Availability of service providers	0.939	0.882	0.937	
5. Accessibility/Convenience	0.724	0.524	0.701	
6. Personal/Manner	0.912	0.832	0.832	
**Variance extracted between dimensions**				**0.707**
**Correlation range**				**0.02–0.546**
**Correlation square range**				**0.000–0.298**

Table 5 also presents the results of discriminant validity analysis, which aims to determine whether the scale factors in our study are distinct from one another. For this assessment, we used the method Fornell and Larcker (1981) suggested [60]. According to this method, discriminant validity is confirmed when the square root of the average extracted variance (AEV) is greater than the highest correlation between any factor and the other factors [60]. In our study, the square root of AEV was calculated to be 0.7, surpassing all correlations, which ranged from 0.02 to 0.546, as well as the correlation squares, which ranged from 0.000 to 0.298, among the factors. Consequently, we can conclude that our scale has established discriminant validity, demonstrating that the factors under investigation are indeed distinct from one another.

#### Reliability

To assess the stability of the scale scores over time, we conducted a test-retest reliability analysis using a sample of 30 participants. In this analysis, we applied Intra Class Correlation (ICC) estimates, utilizing a one-way random-effects model. All scale items exceeded the acceptable threshold of 0.70, indicating a consistent level of reliability of scale scores over time. A comprehensive breakdown of the test-retest results is available in Table 6.

**Table 6:** Results from test-retest reliability and internal consistency of *Section 2*.

Item	*Section 2* dimensions & items	Testing n = 30	Re-testing n = 30	ICC	Cronbach’s alpha
		mean (sd)	mean(sd)		
	**1. Efficacy/Outcome**				0.9
7	Satisfaction with the amount of information provided about the child’s hearing condition.	3.88(0.833)	3.80(0.866)	0.945	
11	The overall effectiveness of therapy.	3.60(1.041)	3.56(1.044)	0.982	
12	Satisfaction with the effect of therapy on the growth of your child’s developmental skills.	3.68(0.852)	3.84(0.850)	0.882	
13	Satisfaction with the rate of developmental skills growth attributable to therapy.	3.64(0.907)	3.76(0.926)	0.883	
14	Satisfaction with the guidance provided by rehabilitation providers on how to continue therapy at home.	3.68(0.748)	3.80(0.764)	0.826	
	**2. Financial aspects**				0.889
15	Satisfaction with the cost of rehabilitation services	3.38(0.970)	3.29(0.955)	0.955	
16	Satisfaction with health insurance services	3.33(1.239)	3.33(1.239)	0.973	
17	Satisfaction with the extent to which rehabilitation expenses are covered by health insurance.	3.39(0.839)	3.43(0.896)	0.971	
	**3. Technical quality**				0.844
5	Satisfaction with the evaluation process pre-implant	4.00(0.816)	4.04(0.790)	0.845	
6	Satisfaction with the timing of the diagnosis of hearing loss and its impact on the developmental skills of the child.	3.68(1.282)	3.56(1.227)	0.962	
8	Satisfaction with the adequacy of information provided about available resources and services for a child with a CI.	3.96(0.889)	3.88(0.833)	0.946	
9	Satisfaction with the act of referring your child to the relevant specialists when necessary.	3.92(0.862)	4.00(0.816)	0.943	
	**4. Availability of service providers**				0.907
1	Satisfaction with the ease of finding rehabilitation providers	2.80(0.957)	2.72(0.792)	0.896	
3	Satisfaction with the ease of locating a rehabilitation clinic/center	2.68(0.900)	2.56(0.870)	0.873	
	5. Accessibility/Convenience				0.833
2	Satisfaction with the ease of making an appointment at the rehabilitation clinic/center	3.00(1.000)	2.96(0.841)	0.883	
4	Satisfaction with the accessibility of the rehabilitation center/clinic or specialist’s location.	2.80(1.190)	2.72(1.137)	0.97	
	**5. Personal/Manner**				-
10	Satisfaction with the consideration and compassion exhibited by rehabilitation providers towards children with CI and their caregivers.	3.64(0.952)	3.72(0.891)	0.953	
	**Scale’s overall internal consistency**				**.855**

Furthermore, Table 6 displays the results of Cronbach’s alpha coefficients, which were utilized to assess the internal consistency of the Likert scale items. The calculated Cronbach alpha values exceeded 0.7. Within each of the scale factors, the internal consistency among items ranged from 0.833 to 0.907. The overall Cronbach’s alpha for the entire scale was 0.855. Results affirm the scale’s overall satisfactory internal consistency.

### Rehabilitation services

#### Rehabilitation services utilization pattern

The findings from *Section 1* descriptive analysis, which focused on “**Utilization patterns of RSs**,” revealed a clear pattern in the RSs received by children with CIs. Speech therapy was the most frequently utilized service, with a significant 81.5% of participants. Audiology services also play a significant role, with 32.6% of children receiving them. In contrast, there is a dramatic underuse of other important services, such as occupational therapy (1.1%) and physical therapy (2.2%), and no mention of any additional RSs.

Turning to the duration of rehabilitation, the data reflects a diverse range of experiences among children with CIs. While 23% of participants received services for a relatively short period of six months or less, the majority displayed varying durations. This included 14.1% receiving services for 7–12 months, 6.3% for 1–2 years, 19.6% for 2.1–3 years, 10.9% for 3.1–4 years, and 9.8% for over 4 years. It’s noteworthy that 6.3% of participants chose not to disclose their rehabilitation duration.

Examining post-rehabilitation progress, the responses reflect the range of outcomes observed by parents. A substantial 38% of parents reported significant improvements in their child’s daily performance. Meanwhile, 32.6% found the progress to be acceptable, and 9.8% noted limited progress. Additionally, 14.1% expressed uncertainty regarding their child’s progress, and 5.4% did not provide a response to this specific question.

#### The extent of parental satisfaction

Within *Section 2*, titled “**Satisfaction Variables**,” parents were asked to express their level of satisfaction with RSs, ranging from "extremely dissatisfied" (ranked 1) to "extremely satisfied" (ranked 5). Interestingly, the mean scores for all items fell within the categories of "neither satisfied nor dissatisfied/neutral" and "satisfied." Notably, items pertaining to the assessment and diagnosis, as well as the intervention and outcomes subsections, received the highest mean scores. This indicates that parents were notably content with these aspects of the RSs. In contrast, the items related to access to CI specialists and facilities received the lowest mean scores. Descriptive analysis of the results is presented in Table 7.

**Table 7:** Descriptive analysis of the Likert scale items and their responses.

	n (%)					Mean (sd)
How satisfied or dissatisfied are you with the following?	Strongly Dissatisfied	Dissatisfied	Neither dis- satisfied nor satisfied	Satisfied	Strongly satisfied	
	1	2	3	4	5	
***Access to rehabilitation providers and facilities***						
1. Ease of finding rehabilitation providers	11(12.0)	24(26.1)	38(41.3)	18(19.6)	1(1.1)	2.72(0.953)
2. Ease of finding a rehabilitation clinic/center	11(12.0)	25(27.2)	41(44.6)	13(14.1)	2(2.2)	2.67(0.939)
3. Ease of making an appointment at the rehabilitation clinic/center	7(7.6)	15(16.3)	48(52.2)	18(19.6)	4(4.3)	2.97(0.919)
4. Convenience of the location of the rehabilitation center/clinic or specialist	9(9.8)	15(16.3)	50(54.3)	16(17.4)	2(2.2)	2.86(0.897)
***Assessment and diagnosis***						
5. The evaluation process pre-implant	6(6.5)	4(4.3)	12(13.0)	45(48.9)	25(27.2)	3.86(1.075)
6. The timing of hearing loss diagnosis in terms of its impact on the child’s developmental skills.	4(4.3)	17(18.5)	6(6.5)	44(47.8)	21(22.8)	3.66(1.151)
7. The amount of information provided about the child’s hearing condition	2(2.2)	13(14.1)	12(13.0)	45(48.9)	17(18.5)	3.70(1.016)
8. The amount of information provided about resources/services available for a child with CI	1(1.1)	13(14.1)	16(17.4)	48(52.2)	12(13.0)	3.63(0.930)
9. Referring to the appropriate specialists when needed	7(7.6)	9(9.8)	10(10.9)	53(57.6)	8(8.7)	3.53(1.066)
10. Consideration and compassion of rehabilitation providers with CI children and their caregivers	8(8.7)	17(18.5)	51(55.4)	16(17.4)	8(8.7)	3.86(1.075)
***Treatment and outcomes***						
11. The effectiveness of therapy in general	2(2.2)	20(21.7)	9(9.8)	38(41.3)	14(15.2)	3.82(0.824)
12. The effectiveness of therapy in the growth of your child’s developmental skills	1(1.1)	14(15.2)	11(12.0)	37(40.2)	20(21.7)	3.51(1.108)
13. The growth rate of developmental skills as a result of therapy	1(1.1)	20(21.7)	13(14.1)	35(38.0)	22(23.9)	3.73(1.049)
14. The guidance received from the rehabilitation providers on how to continue therapy at home.	1(1.1)	9(9.8)	13(14.1)	52(56.5)	17(18.5)	3.63(1.112)
***Financial considerations***						
15. The cost of rehabilitation services	13(14.1)	19(20.7)	20(21.7)	30(32.6)	8(8.7)	3.82(0.889)
16. Health insurance services	8(8.7)	13(14.1)	8(8.7)	36(39.1)	9(9.8)	3.01(1.222)
17. The percentage of rehabilitation expenses covered by health insurance	11(12.0)	16(17.4)	6(6.5)	37(40.2)	3(3.3)	3.34(1.219)

#### Variables associated with overall parental satisfaction

A Pearson correlation analysis has been conducted to determine the degree of correlation (r) between the scale dimensions and the overall parental satisfaction with RSs. The findings revealed a range of R-values, ranging from a minimal 0.02 to a substantial 0.54. Significant correlations were particularly notable in the case of dimension 3, which is related to "technical quality." This dimension comprises critical aspects such as the competence of service providers and their commitment to adhering to best diagnostic and treatment practices. Subsequently, dimension 1, referred to as "efficacy/outcome," emerged as another significant factor. This dimension pertains to how patients perceive the effectiveness of the care they receive in either maintaining or improving their condition during or after receiving rehabilitation. These factors exhibited a clearer influence on parental satisfaction with RSs than other factors. For a more detailed breakdown of the correlation results, please refer to Table 8.

**Table 8:** Results from Pearson correlation between the satisfaction dimensions and the overall satisfaction with RSs.

Dimension #	Satisfaction dimensions	r-value	Sig.
1	Efficacy/ outcome	.48**	0.001
2	Financial aspects	0.12	0.286
3	Technical quality	.54**	0.001
4	Availability of service providers	0.11	0.295
5	Accessibility/convenience	0.02	0.837
6	Interpersonal manner	0.08	0.442

**. Correlation is significant at the 0.01 level (2-tailed).

#### Parents’ suggestions to enhance satisfaction

In response to an open-ended question designed to identify the priorities of parents of children with CIs to enhance their satisfaction with RSs, 38% (35 out of 92) of participants offered insightful feedback, which we have categorized into several key factors:

*Financial support*. A significant majority, comprising 59% (21 respondents), emphasized the urgent need for a substantial reduction in therapy session costs. Additionally, 41% (14 respondents) strongly advocated for health insurance options tailored specifically to the needs of CI users.

*Enhanced accessibility and monitoring of services*. Parents highlighted the importance of improving service accessibility, with approximately 35% (12 respondents) emphasizing the need to increase the availability of CI rehabilitation clinics. Rigorous monitoring of the services provided by these facilities was also deemed crucial for maintaining the quality and effectiveness of rehabilitation. Furthermore, 45% (16 respondents) stressed the importance of establishing rehabilitation centers across the country, addressing geographical challenges, and reducing the travel burden for many families, thereby ensuring accessibility for all.

*Improved service delivery and outcome*. For improved service delivery and outcome, roughly 29.5% (10 respondents) requested more frequent therapy sessions and the introduction of online therapy options to ensure more flexibility and efficacy of services. An additional 30% (11 respondents) called for the implementation of a newborn hearing screening policy. Such a policy would not only enable early diagnosis but also improve overall rehabilitation outcomes by ensuring timely and effective interventions for children with CIs.

*Specialized education*. Recognizing the unique needs of CI users, 23.5% (8 respondents) emphasized the significance of establishing specialized schools dedicated to children with CIs. Moreover, 29% (10 respondents) highlighted the importance of promoting the inclusion of these children in general education classrooms and providing specialized training for teachers to effectively educate and support them. An additional 32.5% (10 respondents) suggested integrating speech therapy services into regular classrooms, a critical step in enhancing the overall educational experience for CI users.

*Raising public awareness*. Approximately 53% of respondents recommended intensifying efforts to increase public awareness of CIs. These efforts could include workshops and training sessions targeting parents, teachers, professionals, and the general public. By increasing knowledge and understanding, society can support and integrate individuals with CIs more effectively.

## Discussion

A survey was devised, piloted, revised, and administered to a larger sample of parents in order to assess their satisfaction with the RSs provided to their children with CIs. Ninety two parents of children with CIs in Jordan evaluated RSs by completing this survey. The instrument surveyed parents about RSs and investigated factors that increase their satisfaction with these services. The results confirmed the scale’s validity and reliability while providing valuable insights into parental perspectives on RSs. Moreover, the study revealed important utilization patterns and offered constructive recommendations for the improvement of CI rehabilitation services in Jordan.

The current research introduces an instrument in Arabic to assess parental satisfaction with RSs. To this end, to ensure that the survey instrument accurately measures the intended construct and that the questions are clear and relevant to the target population, we established content validity through the involvement of experts in the design phase and conducted cognitive interviews. This methodology is in line with best practices for establishing content validity [52, 64].

We demonstrated the construct validity of the scale questions in *Section 2* of the survey using exploratory factor analysis (EFA) [65]. The Kaiser-Meyer-Olkin (KMO) measure and Bartlett’s Test of Sphericity [66, 67] measures supported the results in demonstrating the suitability of the data for factor analysis. EFA results suggested a six-factor solution with 17 items. The majority of the items had factor weights greater than 0.5 [59]. The six factors were: 1) efficiency and outcome; 2) financial aspects; 3) technical quality; 4) service provider and facility availability; 5) service accessibility and convenience; and 6) interpersonal manner. These six factors nearly encompass the most significant aspects of patient satisfaction with care services identified in prior research. Specifically, interpersonal manner, technical quality, accessibility/convenience, and financial aspects are by far the most frequently measured dimensions of care [7]. Hall and Doman’s research findings indicated that patients expressed the highest levels of satisfaction in areas related to overall quality, compassion, competency, and the resulting outcomes [61]. Conversely, factors such as cost, bureaucratic processes (including waiting times), and the attention given to psychosocial issues received the lowest satisfaction ratings. Consistent with previous research, the results endorse the applicability of these factors in evaluating satisfaction among Arabic-speaking parents, as they encompass crucial elements of parental satisfaction with RSs. These findings offer valuable insights for healthcare providers and policymakers, guiding efforts to improve care quality and meet the diverse needs of families with children who have CIs.

To assess the scale’s reliability, we followed the Intra-Class Correlation (ICC)-recognized procedure for test-retest reliability [62]. The results, with all scale items exceeding the acceptable threshold of 0.70, demonstrate a consistent level of reliability in the scale scores over time. Also, the high Cronbach’s alpha coefficients from internal consistency analysis [68] within each scale factor and the overall satisfactory internal consistency (Cronbach’s alpha = 0.855) further affirm the reliability of the questionnaire.

Our survey results echoed findings from previous studies showing that speech therapy was the most commonly utilized RS, confirming its well-established significance in fostering language and communication skills development in children with CIs [20, 69]. Audiology services followed closely, reaffirming their critical role in optimizing CI performance [20, 70]. However, there is a concerning trend of underutilization of other crucial services, such as physical therapy, occupational therapy, and psychology. This underutilization brings attention to a potential gap in comprehensive care, despite the essential role these services play in addressing the broader developmental needs of children with CIs [71–73]. The findings highlight the need for a more multidisciplinary approach to address the holistic developmental needs of these children.

In terms of rehabilitation duration, it’s worth noting that more children received services for six months or less than for longer periods of time, which is in line with the timeframe for aural rehabilitation that the government’s initiative supports for CIs. However, it’s essential to acknowledge that this six-month duration may not be enough, even with early intervention for HL. Research indicates that the brain’s plasticity is most pronounced in early childhood, and ideally, rehabilitation should continue until children with CIs reach their maximum potential in speech and language skills, typically by age three or school entry [74, 75]. Extensive studies show that both rehabilitation outcomes and parental satisfaction improve with longer intervention periods length [24]. This highlights the importance of extending and customizing rehabilitation services to ensure that each child achieves their full communication potential, ideally until school entry.

Interestingly, the survey results demonstrate that parents generally hold a positive view of RSs for children with CIs, with particular satisfaction with assessment and diagnosis, as well as intervention and outcome aspects of services. This degree of satisfaction emphasizes the critical role that precise assessment and effective intervention play in the overall success of CI programs, aligning with established research in the field [76]. This positive parental perception of RSs might be influenced by the significant attention that the Jordanian health sector, government, and media have devoted to the CI process. Government initiatives, specifically Hearing Without Borders, aimed at providing medical and rehabilitation support and post-surgery training for children with CI [44], as well as media coverage highlighting the importance of CIs [77], have likely contributed to this satisfaction. This suggests that the coordinated efforts of the Jordanian health sector, government, and media to promote awareness and provide essential support can have a significant impact on positive parental perceptions of RSs.

In contrast, RSs related to access to CI specialists and facilities received the lowest mean scores. Given Jordan’s scarcity of trained professionals with expertise in CI rehabilitation, the decline in satisfaction with service accessibility is understandable. This factor, along with the limited number of CI-specialized clinics and facilities primarily situated in Amman, the capital city of Jordan, may lead to these results. Even in developed countries, accessing RSs can be a challenge, particularly for rural residents [36]. These findings highlight potential areas for improvement in terms of accessibility to specialized care and facilities.

The Pearson correlation analysis provided further insight into the factors influencing parental satisfaction with RSs. Significantly, both "technical quality," which encompasses the expertise of service providers and their adherence to best practices, and "efficacy/outcome," reflecting parents’ perceptions of the care’s effectiveness in improving their child’s condition, were found to have strong and positive correlations with overall parental satisfaction regarding RSs. These findings are consistent with earlier research highlighting the critical role of skilled professionals in the CI rehabilitation process [78] and highlight the importance of favorable treatment outcomes in shaping parental satisfaction [79].

Last, the study asked parents of children with CIs to identify their priorities for improving satisfaction with RSs. The results were comparable to those of previous studies of developing countries [39]. Parents highlighted key factors, including the need for financial support, reduced therapy costs, and specialized health insurance options, to alleviate the financial burden on families. Additionally, addressing accessibility issues through the establishment of more CI rehabilitation clinics, nationwide centers, and rigorous quality control was emphasized. Suggestions for enhancing service delivery included more frequent therapy sessions, online options, implementing newborn hearing screening, and specialized education, with an emphasis on early intervention and individualized educational approaches. Lastly, raising public awareness through workshops and training was seen as crucial. The findings align with previous research on the needs of children with HL [80]. These findings highlight the multifaceted difficulties faced by parents and the need for comprehensive support systems.

This study comes with several limitations that warrant acknowledgment. Firstly, our participant sample was drawn conveniently from Amman, Jordan’s capital, and its surrounding governorates. While this approach was practical, a more representative sample could have been achieved if there were a readily accessible data bank of CI users from which participants could be randomly selected. Secondly, although the intended sample size for this study was considered sufficient, a larger sample size could have potentially impacted the significance of the results, offering greater generalizability. Thirdly, the absence of standardized satisfaction evaluation measures specifically validated for families of children with CIs posed a limitation. A validated comparison of our tool’s results with those obtained from another standardized instrument could have increased the reliability of our findings. Unfortunately, due to the lack of such standardized assessment tools in Arabic, we were unable to pursue this avenue. Thus, in order to improve the validity and reliability of our assessments, it is crucial that we develop valid instruments that are tailored to Arabic-speaking participants for future research endeavors.

## Supporting information

S1 Data (XLS)

S1 Checklist STROBE statement—Checklist of items that should be included in reports of observational studies. (DOCX)
