# Supplementary material for: Reliability and validity assessment of a survey: Measuring satisfaction with cochlear implant rehabilitation services for children in Jordan
Source: PLoS One. 2023 Dec 18;18(12):e0295939. doi: 10.1371/journal.pone.0295939 (PMC10727441; doi:10.1371/journal.pone.0295939)
Supplement: S1 Checklist — (DOCX) [file pone.0295939.s002.docx]

STROBE Statement—checklist of items that should be included in reports of observational studies

|  | Item No. | Recommendation | Page  No. | Relevant text from manuscript |
| --- | --- | --- | --- | --- |
| **Title and abstract** | 1 | (*a*) Indicate the study’s design with a commonly used term in the title or the abstract | p.2 | Reliability and validity assessment of a survey: Measuring satisfaction with cochlear implant rehabilitation services for children in Jordan |
|  |  | (*b*) Provide in the abstract an informative and balanced summary of what was done and what was found | p.2-3 | The study included 92 participants and followed a four-step methodology: 1) a literature review and expert input; 2) cognitive interviews, pilot testing, and test-retest reliability testing; 3) data collection; 4) validity and reliability assessments.  The survey's validity was confirmed. Expert input and cognitive interviews improved content validity, and factor analysis established construct validity by revealing six factors explaining 82.33% of the variance in the survey scale. Convergent and discriminant validity were confirmed (composite reliability >0.7 and average variance extracted value >0.5). Cronbach's α exceeded 0.8 for each factor and reached 0.855 for the total scale. Survey results showed reliance on speech therapy and audiology, varied rehabilitation durations, and progress. Parents expressed overall satisfaction, particularly influenced by technical quality and efficacy/outcome dimensions. Parents' recommendations to enhance satisfaction with RSs included financial support, improved service accessibility, enhanced service delivery, specialized education, and increased public awareness.  This study validates an Arabic satisfaction survey, emphasizing the significance of multidisciplinary, extended rehabilitation programs, skilled professionals, and positive outcomes. It emphasizes the necessity for improved access to specialized care and collaboration among healthcare, government, and media to shape parental perceptions of RSs. While the findings indicate overall satisfaction, they also reveal challenges faced by parents, highlighting the need for comprehensive support systems. These insights assist healthcare providers and policymakers in enhancing care quality and meeting the needs of CI children’s families, thereby improving the RSs experience in Jordan. |
| Introduction | | | |  |
| Background/rationale | 2 | Explain the scientific background and rationale for the investigation being reported | p.4 | Hearing loss is the world's fourth leading cause of disability. Without access to early diagnosis and appropriate intervention or rehabilitation, children with hearing loss may have lifelong deficits in speech and language acquisition, poor academic performance, social maladjustments, and emotional difficulties. Therefore, having an effective healthcare system that considers the entire spectrum of care, including the diagnosis and rehabilitation of hearing loss, as well as thoroughly evaluating that system to enhance the quality of care, is crucial. |
| Objectives | 3 | State specific objectives, including any prespecified hypotheses |  | There is a notable absence of existing rehabilitation assessment questionnaires that have been applied specifically to Arabic-speaking parents of children with CIs. To address this gap, we developed and validated a survey instrument tailored exclusively for this study's purpose. This study has two main objectives: Firstly, it aims to validate the satisfaction scale integrated into the survey. Secondly, it seeks to investigate parental satisfaction with RSs for children with CIs in Jordan, focusing on four key objectives:  • To explore the RSs utilization patterns by children with CIs in Jordan.  •To determine the extent to which parents of children with CIs in Jordan express satisfaction with the RSs they receive.  •To determine which specific variables related to satisfaction are most strongly correlated with the overall satisfaction of parents of children with CIs with RSs.  •To highlight parents' suggestions on how to enhance their satisfaction with RSs for children with CIs. |
| Methods | | | |  |
| Study design | 4 | Present key elements of study design early in the paper | p. 9-12 | An Arabic survey was designed to evaluate parents’ satisfaction with RSs. To do so, four steps were carried out: (1) a literature review and the design of the survey instrument; (2) cognitive interviews, piloting, and reliability testing; (3) determining sample size and construct validity testing; and (4) analysis of the evidence of convergent and discriminant validity. The stages are described below. |
| Setting | 5 | Describe the setting, locations, and relevant dates, including periods of recruitment, exposure, follow-up, and data collection | p.12-13 | Participants were recruited from various sources, including public speech and hearing clinics in hospitals and universities, private speech and hearing clinics, special education centers, and through personal contacts made by the researchers.  The study was approved by the Research and Ethics Committee at the University of Jordan. All the participants consented to participate in the study by signing informed consent form and completing and returning the survey. All methods were carried out in accordance with relevant guidelines and regulations. The survey was distributed alongside a cover letter that outlined the study's objectives, stressed the voluntary nature of participation, and guaranteed the confidentiality of all personal information and opinions collected. To ensure anonymity, each participant was assigned a unique survey code, and they had the flexibility to complete the survey either during their facility visit or on a subsequent visit. Reminders and replacement copies were provided for those who didn't return the survey. Participants were instructed to contact the study's principal investigator via email or phone if they had any questions. Data collection spanned six months. |
| Participants | 6 | (*a*) *Cohort study*—Give the eligibility criteria, and the sources and methods of selection of participants. Describe methods of follow-up  *Case-control study*—Give the eligibility criteria, and the sources and methods of case ascertainment and control selection. Give the rationale for the choice of cases and controls  *Cross-sectional study*—Give the eligibility criteria, and the sources and methods of selection of participants | p. 12 | A non-probability convenience sampling method was utilized in data collection. This approach is commonly used in exploratory research to investigate the attitudes and perspectives of participants. The inclusion criteria involved parents of congenital prelingually deafened children who had received CIs and had experience with RSs. Additionally, participants needed to be proficient in reading and writing standard Arabic. Excluded from the study were parents of children who had received CIs in Jordan but were receiving RSs in another country at the time of the study.  Of the 200 surveys that were sent out, 100 (50%) were returned, and 92 (46%) were used in the analysis. It's worth noting that eight surveys had to be excluded from the analysis due to significant missing data. The participants who chose not to return the survey cited reasons such as changing their minds about study participation, switching therapy facilities, or discontinuing therapy. |
|  |  | (*b*) *Cohort study*—For matched studies, give matching criteria and number of exposed and unexposed  *Case-control study*—For matched studies, give matching criteria and the number of controls per case |  |  |
| Variables | 7 | Clearly define all outcomes, exposures, predictors, potential confounders, and effect modifiers. Give diagnostic criteria, if applicable | p.9-10 | The primary objective of the study is to assess the validity and reliability of the instrument developed specifically for this research.  Then use to survey to explore the RSs utilization patterns, the extent to which parents of children with CIs express satisfaction with RSs, and to explore the variables that are correlated with the overall satisfaction of parents of children with CIs with RSs. |
| Data sources/ measurement | 8* | For each variable of interest, give sources of data and details of methods of assessment (measurement). Describe comparability of assessment methods if there is more than one group | *p.17-21*  *21-22* | To establish the survey's validity, content and construct testing were conducted. Validity was further scrutinized through cognitive interviews, as well as by administering factor analysis and analyses of convergent and discriminant validity on the survey.  The reliability of the survey was evaluated through an examination of test-retest reliability and testing the internal consistency of the survey’s Likert scale items.  Descriptive analysis was used to explore the RSs utilization patterns, and the extent to which parents of children with CIs express satisfaction with RSs.  A Pearson correlation analysis has been conducted to determine the degree of correlation (r) between the scale dimensions and the overall parental satisfaction with RSs. |
| Bias | 9 | Describe any efforts to address potential sources of bias | p.6 | To diversify data collection and minimize participant selection bias commonly found in convenience sampling, the researchers recruited the participants through different CI clinics, private speech and hearing clinics, special education centers, and personal contacts over different days and times. That, and worked on recruiting as many participants as possible. |
| Study size | 10 | Explain how the study size was arrived at | p.11-13 | Children with CIs are dispersed throughout Jordan, and no information repository exists for them. Therefore, locating these participants is not easy. According to the aforementioned information, approximately 1,000 children received their CIs in Jordan. Consequently, 100 participants were considered a good maximum sample size for this study. Most statisticians agree that a decent survey sample size of a population is 10%, as long as this does not exceed 1000 (18). The questionnaire was sent out to 200 participants. Out of the 200 parents contacted in October 2022, 110 (55%) returned the questionnaire. Of the 200 surveys that were sent out, 100 (50%) were returned, and 92 (46%) were used in the analysis. It's worth noting that eight surveys had to be excluded from the analysis due to significant missing data. The participants who chose not to return the survey cited reasons such as changing their minds about study participation, switching therapy facilities, or discontinuing therapy. |

Continued on next page

| Quantitative variables | 11 | Explain how quantitative variables were handled in the analyses. If applicable, describe which groupings were chosen and why | p. 24 | In response to an open-ended question designed to identify the priorities of parents of children with CIs to enhance their satisfaction with RSs, 38% (35 out of 92) of participants offered insightful feedback, which we have categorized into several key factors:  Financial Support:  Enhanced Accessibility and Monitoring of Services:  Improved Service Delivery and Outcome:  Specialized Education:  Raising Public Awareness: |
| --- | --- | --- | --- | --- |
| Statistical methods | 12 | (*a*) Describe all statistical methods, including those used to control for confounding | P.17-22 | To establish the survey's validity, content and construct testing were conducted. Validity was measured by administering factor analysis and analyses of convergent and discriminant validity on the survey.  The reliability of the survey was evaluated through an examination of test-retest reliability and testing the internal consistency of the survey’s Likert scale items.  Descriptive analysis was used to explore the RSs utilization patterns, and the extent to which parents of children with CIs express satisfaction with RSs.  A Pearson correlation analysis has been conducted to determine the degree of correlation (r) between the scale dimensions and the overall parental satisfaction with RSs. |
|  |  | (*b*) Describe any methods used to examine subgroups and interactions |  | NA |
|  |  | (*c*) Explain how missing data were addressed | P.12 | Eight surveys had to be excluded from the analysis due to significant missing data. |
|  |  | (*d*) *Cohort study*—If applicable, explain how loss to follow-up was addressed  *Case-control study*—If applicable, explain how matching of cases and controls was addressed  *Cross-sectional study*—If applicable, describe analytical methods taking account of sampling strategy |  |  |
|  |  | (*e*) Describe any sensitivity analyses |  |  |
| Results | | | | |
| Participants | 13* | (a) Report numbers of individuals at each stage of study—eg numbers potentially eligible, examined for eligibility, confirmed eligible, included in the study, completing follow-up, and analysed |  |  |
|  |  | (b) Give reasons for non-participation at each stage |  |  |
|  |  | (c) Consider use of a flow diagram |  |  |
| Descriptive data | 14* | (a) Give characteristics of study participants (eg demographic, clinical, social) and information on exposures and potential confounders | p. 15 & 16 | Participants’ characteristics are presented on Tables 2 and 3 |
|  |  | (b) Indicate number of participants with missing data for each variable of interest |  |  |
|  |  | (c) *Cohort study*—Summarise follow-up time (eg, average and total amount) |  |  |
| Outcome data | 15* | *Cohort study*—Report numbers of outcome events or summary measures over time |  |  |
|  |  | *Case-control study—*Report numbers in each exposure category, or summary measures of exposure |  |  |
|  |  | *Cross-sectional study—*Report numbers of outcome events or summary measures | p. 18  p.19  p.20  p.23  p.23 | Table 4. Items in Section 2 loadings on the satisfaction dimensions, as determined through principal component analysis.  Table 5. Convergent and discriminant validity analysis results  Table 6. Results from test-retest reliability and internal consistency  Table 7. Descriptive analysis of the Likert scale items and their responses  Table 8. Results from Pearson correlation between the satisfaction dimensions and the overall satisfaction with RSs |
| Main results | 16 | (*a*) Give unadjusted estimates and, if applicable, confounder-adjusted estimates and their precision (eg, 95% confidence interval). Make clear which confounders were adjusted for and why they were included |  | NA |
|  |  | (*b*) Report category boundaries when continuous variables were categorized |  |  |
|  |  | (*c*) If relevant, consider translating estimates of relative risk into absolute risk for a meaningful time period |  |  |

Continued on next page

| Other analyses | 17 | Report other analyses done—eg analyses of subgroups and interactions, and sensitivity analyses |  |  |
| --- | --- | --- | --- | --- |
| Discussion | | | | |
| Key results | 18 | Summarise key results with reference to study objectives | P.16-24 | The main results of the study confirmed the validity of the survey. Expert input and cognitive interviews improved content validity, and factor analysis established construct validity by revealing six factors explaining 82.33% of the variance in the survey scale. Convergent and discriminant validity were confirmed (composite reliability >0.7 and average variance extracted value >0.5). Cronbach's α exceeded 0.8 for each factor and reached 0.855 for the total scale. Survey results showed reliance on speech therapy and audiology, varied rehabilitation durations, and progress. Parents expressed overall satisfaction, particularly influenced by technical quality and efficacy/outcome dimensions. Parents' recommendations to enhance satisfaction with RSs included financial support, improved service accessibility, enhanced service delivery, specialized education, and increased public awareness. |
| Limitations | 19 | Discuss limitations of the study, taking into account sources of potential bias or imprecision. Discuss both direction and magnitude of any potential bias | p.29 | This study comes with several limitations that warrant acknowledgment. Firstly, our participant sample was drawn conveniently from Amman, Jordan’s capital, and its surrounding governorates. While this approach was practical, a more representative sample could have been achieved if there were a readily accessible data bank of CI users from which participants could be randomly selected. Secondly, although the intended sample size for this study was considered sufficient, a larger sample size could have potentially impacted the significance of the results, offering greater generalizability. Thirdly, the absence of standardized satisfaction evaluation measures specifically validated for families of children with CIs posed a limitation. A validated comparison of our tool's results with those obtained from another standardized instrument could have increased the reliability of our findings. Unfortunately, due to the lack of such standardized assessment tools in Arabic, we were unable to pursue this avenue. Thus, in order to improve the validity and reliability of our assessments, it is crucial that we develop valid instruments that are tailored to Arabic-speaking participants for future research endeavors. |
| Interpretation | 20 | Give a cautious overall interpretation of results considering objectives, limitations, multiplicity of analyses, results from similar studies, and other relevant evidence |  | The study successfully developed a valid survey to assess parental satisfaction with Cochlear Implant (CI) rehabilitation services in Jordan. Valuable insights into utilization patterns and key improvement areas were gained. However, caution is needed due to limitations such as a non-representative sample and absence of standardized measures. Findings align with previous studies but should be interpreted carefully, considering the study's objectives and the need for future research with larger, diverse samples and refined measurement tools to enhance validity and generalizability. Overall, while providing valuable insights, the study's results require cautious interpretation and further investigation. |
| Generalisability | 21 | Discuss the generalisability (external validity) of the study results | 29 | The generalizability (external validity) of the study results is somewhat limited. The sample, drawn mainly from Amman and surrounding areas, may not fully represent the diversity of the entire Jordanian population, particularly in more rural regions. The sample size, although deemed sufficient for the study's objectives, could have been larger and more diverse to enhance external validity. Cultural factors specific to Jordan and variations in healthcare systems across countries may impact the applicability of the findings to other populations. Additionally, the survey instrument being in Arabic and tailored to the Jordanian context may limit its use in different Arabic-speaking regions. To improve generalizability, future research should include more diverse samples, consider cross-cultural validation, and account for healthcare system differences. |
| Other information | |  | | |
| Funding | 22 | Give the source of funding and the role of the funders for the present study and, if applicable, for the original study on which the present article is based |  | This study was not funded by any grant. |

*Give information separately for cases and controls in case-control studies and, if applicable, for exposed and unexposed groups in cohort and cross-sectional studies.

**Note:** An Explanation and Elaboration article discusses each checklist item and gives methodological background and published examples of transparent reporting. The STROBE checklist is best used in conjunction with this article (freely available on the Web sites of PLoS Medicine at http://www.plosmedicine.org/, Annals of Internal Medicine at http://www.annals.org/, and Epidemiology at http://www.epidem.com/). Information on the STROBE Initiative is available at www.strobe-statement.org.
